# Estimation of gait parameters in healthy and hemiplegic individuals using Azure Kinect: a comparative study with the optoelectronic system

**DOI:** 10.3389/fbioe.2024.1449680

**Published:** 2024-11-25

**Authors:** Serena Cerfoglio, Claudia Ferraris, Luca Vismara, Gianluca Amprimo, Lorenzo Priano, Matteo Bigoni, Manuela Galli, Alessandro Mauro, Veronica Cimolin

**Affiliations:** ^1^ Department of Electronics, Information and Bioengineering, Politecnico di Milano, Milan, Italy; ^2^ Division of Neurology and Neurorehabilitation - IRCCS Istituto Auxologico Italiano, Verbania, Italy; ^3^ Institute of Electronics, Computer and Telecommunication Engineering (IEIIT), Consiglio Nazionale delle Ricerche (CNR), Turin, Italy; ^4^ Department of Control and Computer Engineering, Politecnico di Torino, Turin, Italy; ^5^ Department of Neurosciences, University of Turin, Turin, Italy

**Keywords:** RGB-D sensors, gait analysis, hemiplegia, markerless motion analysis, hemiplegic individuals

## Abstract

**Introduction:**

Walking ability is essential for maintaining functional independence, but it can be impaired by conditions like hemiplegia resulting from a stroke event. In post-stroke populations, accurately assessing gait anomalies is crucial for rehabilitation to promote functional recovery, and to prevent falls or injuries.

**Methods:**

The aim of this study is to evaluate gait-related parameters using a solution based on a single RGB-D camera, specifically Microsoft Azure Kinect DK (MAK), on a short walkway in both healthy (n= 27) and post-stroke individuals with hemiplegia (n= 20). The spatio-temporal and center of mass (CoM) parameters estimated by this approach were compared with those obtained from a gold standard motion capture (MoCap) system for instrumented 3D gait analysis.

**Results:**

The overall findings demonstrated high levels of accuracy (> 93%), and strong correlations (r > 0.9) between the parameters estimated by the two systems for both healthy and hemiplegic gait. In particular, some spatio-temporal parameters showed excellent agreement in both groups, while CoM displacements exhibited slightly lower correlation values in healthy individuals.

**Discussion:**

The results of the study suggest that a solution based on a single optical sensor could serve as an effective intermediate tool for gait analysis, not only in clinical settings or controlled environments but also in those contexts where gold standard systems are not feasible.

## 1 Introduction

Level walking is a major component of daily physical activity, playing a key role in ensuring full functional independence and contributing significantly to overall health (Cimolin and Galli, 2014). Gait abilities depend on the coordination between the central nervous and the musculoskeletal systems, which can be disrupted by pathological states, such as Parkinson’s disease and hemiplegia (Moon et al., 2016; Snijders et al., 2011). Hemiplegia is a condition characterized by the deficit of voluntary motor function on one side of the body, and it represents one of the most common impairments in stroke survivors that significantly impacts a variety of motor skills. Specifically, it manifests as muscular weakness or hemi-paralysis, affecting both arm function and locomotor abilities (Chen et al., 2005; Gowland et al., 1992). With respect to gait, hemiplegic patterns are characterized by asymmetry and foot dragging due to the weakness of the affected side, together with aberrant torso tilting and rotation (Beyaert et al., 2015; Moon et al., 2016; Saccani et al., 2022; Wonsetler and Bowden, 2017).

Rehabilitation plays a key role in the overall functional recovery of post-stroke individuals. Several studies have shown that the intensity, frequency, and specific stimuli of the rehabilitation protocol can contribute to the improvement and recovery of motor and cognitive functions, as well as ecological tasks that mimic everyday behaviors and actions (Dobkin, 2005; Rosbergen et al., 2016). However, a quantitative analysis and evaluation of patient performance could also support the evidence for the clinical effectiveness of rehabilitation and physical training treatments through objective measures. Concerning walking, which is a pillar in many rehabilitation protocols, accurate and objective monitoring of improvement or decline in walking patterns is essential from the perspective of designing and evaluating dedicated rehabilitation protocols that are tailored to each patient’s condition (Langhorne et al., 2011), thus promoting improvement in locomotion skills and perceived safety in daily activities.

Three-dimensional (3D) marker-based optoelectronic motion capture systems (MoCap) represent the gold standard for assessing either normal or pathological gait. MoCap systems accurately provide objective information about joint motion (i.e., kinematics), time-distance variables (i.e., spatio-temporal parameters), and joint moments and powers (i.e., kinetics) if used together with force plates. Data retrieved from MoCap allow for a comprehensive and quantitative assessment of gait patterns allowing for functional performance analysis and the identification of atypical features, as well as for determining the level of functional impairment associated to a pathological condition. However, despite their acknowledged accuracy and consistency, some factors limit the applicability of MoCap systems in daily clinical practice, including high costs, the need for specifically designed laboratory environments with trained technical staff, as well as the need of wearing minimal clothing which can be an issue for people with dysfunction in dressing (Bugané et al., 2012; Colyer et al., 2018; van den Noort et al., 2013).

In recent years, new emerging technologies based either on wearable sensors (e.g., inertial measurement units (IMUs) and accelerometers) and optical sensors (e.g., RGB, Depth, and RGB-Depth cameras) have been proposed as cost-effective and handy alternative tools to MoCap for the analysis and evaluation of several motor skills. In particular, optical sensors supported by body-tracking capabilities such as the Microsoft Kinect^®^ have shown promise for motion capture and analysis across various tasks, conditions, and purposes (Clark et al., 2019; González-Ortega et al., 2014; Milosevic et al., 2020; Mousavi Hondori and Khademi, 2014; Muro-de-la-Herran et al., 2014). Originally designed for entertainment purposes, the Microsoft Kinect^®^ has evolved into a pioneering vision-based motion capture system whose applications now extend to medical, clinical, and rehabilitation fields (Asaeda et al., 2018; Cerfoglio et al., 2022; Pedraza-Hueso et al., 2015). Thanks to its innovative technology exploiting the integration of colour and depth sensors, the device operates by automatically estimating the real-time position of the major joints through some anatomical landmarks provided by a 3D skeletal model (Albert et al., 2020; Wang et al., 2015). Solutions based on this kind of device do not require a dedicated laboratory and complex setup like traditional MoCap systems. In addition, they allow for non-intrusive motion tracking, as they require neither preliminary subject preparation (i.e., the applications of markers on the body based on biomechanical models), which can often be troublesome for patients with functional limitations, nor dedicated handheld controllers (Liao et al., 2018). Thanks to their versatility, these approaches have also opened new perspectives for motion analysis and monitoring especially in unsupervised and remote settings, including private home environments by enabling continuous motoring of motor skills in real-life scenarios (Anton et al., 2018; Da Gama et al., 2015; Ferraris et al., 2024; Stone and Skubic, 2015). Despite the strengths mentioned, there is a need to verify the accuracy and robustness of these solutions in tracking human body movements. For this purpose, validation procedures and comparisons with gold standard MoCap systems are essential.

Regarding Kinect-based solutions, several studies have investigated the accuracy on gait analysis data in both healthy individuals and pathological conditions using Microsoft Xbox Kinect or Microsoft Kinect v2, the older device models (Buongiorno et al., 2019; Mortensen et al., 2015; Palacios-Navarro et al., 2015; Vilas-Boas et al., 2019). With respect to healthy individuals, various studies highlighted the reliability in estimating gait-related metrics, showing good concurrent validity with the traditional MoCap systems (Clark et al., 2013; Dubois and Bresciani, 2018; Eltoukhy et al., 2017; Pfister et al., 2014; Rocha et al., 2015). In particular, spatio-temporal parameters estimated from these Kinect models have been reported to strongly correlate with the ones computed by MoCap systems, although a lack of accuracy has been shown in estimating short temporal phases, such as foot off and step time (Xu et al., 2015). Similar findings have also been observed in clinical populations, such as post-stroke individuals. For instance, Ferraris et al. (2021) reported strong consistency, accuracy, and correlation with the gold standard for gait spatio-temporal gait parameters, thus suggesting the reliability of the Kinect v2 model in detecting and evaluating gait impairments. Moreover, good agreement levels have also been reported for parameters related to the body’s center of mass (CoM), which could be relevant in identifying specific gait alterations associated with increased risk of fall, such as lateral body sway (Johansson et al., 2019; Yanohara et al., 2014).

Regarding Microsoft Azure Kinect DK (MAK), the latest device model, several studies have verified and validated it versus the previous models (Kurillo et al., 2022; Ma et al., 2020; Tölgyessy et al., 2021), demonstrating its superior performance in terms of on-board sensors and body tracking capabilities (Albert et al., 2020; Yeung et al., 2021) as well as its suitability for motion analysis in different tasks, such as gait, posture, and sit to stand (Amprimo et al., 2022; Antico et al., 2021; Guess et al., 2022; Thomas et al., 2022) even compared to gold reference systems.

Focusing on gait analysis, a few studies in the literature (Bertram et al., 2023; Guess et al., 2022; Ripic et al., 2022) have investigated the accuracy of Microsoft Azure Kinect DK on healthy subjects compared with a gold standard MoCap system, proposing a single-camera solution. In the study by Guess et al. (2022), some spatio-temporal parameters of overground walking (stride length, stride time, step length, and step width) were simultaneously collected by MAK and MoCap systems on twenty young healthy participants. The study demonstrated high values of correlation coefficients between the two systems for all spatio-temporal parameters investigated. Bertram et al. (2023) looked at other kinematic parameters (gait speed, step length, cadence, step time, and arm mobility features) in addition to assessing other motor tasks (balance, getting up and sitting down, walking in place). The MAK showed high to excellent relative and absolute agreement for spatial and temporal measurements, although the gait results were affected by some interference with the gold standard systems, probably caused by the excessive proximity of the infrared emitters of the optoelectronic system. In contrast, Ripic et al. (2022) focused the analysis on gait kinetics, demonstrating high correlations between MAK and gold standard measurements, and superior accuracy of MAK compared to previous models. These results suggest that a single MAK sensor can provide clinically relevant measurement of spatio-temporal parameters during gait. Despite these encouraging results, however, further investigation is necessary by extending the analysis to other gait parameters and locomotion features, which are traditionally considered clinically relevant to highlight gait disorders in pathological conditions (Perry and Burnfield, 2010).

With this in mind, the present study aims to extend the comparison between a single MAK-sensor solution and a gold standard (i.e., an optoelectronic system) in terms of traditional spatio-temporal gait parameters by considering both healthy and post-stroke hemiplegic individuals. In addition, for a more comprehensive analysis of the post-stroke condition, the comparison also includes some metrics related to body CoM displacements during walking. Indeed, this is an interesting and typical feature of walking disorders in post-stroke survivors with hemiplegia caused by the greater difficulty in adequately counterbalancing the muscle work of the affected and unaffected limbs (do Carmo et al., 2015; Iida and Yamamuro, 1987; Tesio and Rota, 2019). In summary, the study aims to cover the following gaps in performance analysis: 1) investigate MAK on post-stroke hemiplegic individuals, since no other comparison studies are available; 2) investigate MAK on additional spatio-temporal parameters from previous studies to provide a more in-depth gait analysis; 3) investigate MAK in estimating CoM displacements during walking, which are particularly relevant in hemiplegic individuals.

The subsequent sections of this paper are organized as follows. Section 2 presents the materials and methods, covering the study design, participant recruitment, experimental setup, data processing for both MAK and MoCap, and statistical analysis. Section 3 presents the results, providing a comprehensive comparison of the two systems and highlighting their agreement in estimating both spatio-temporal and center of mass parameters, along with statistical measures and metrics. In Section 4, we discuss the findings, study limitations, and their implications for clinical practice. Finally, Section 5 summarizes the main contributions of this work and suggests directions for future research.

## 2 Materials and methods

### 2.1 Participants

Two cohorts of subjects were enrolled on a voluntary basis. The first group included post-stroke individuals recruited among the patients admitted to San Giuseppe Hospital (IRCCS Istituto Auxologico Italiano, Piancavallo, Italy), whilst the second group included healthy individuals recruited among the hospital’s staff. Inclusion criteria for the post-stroke group were the following: age ≥18 years, presence of unilateral hemiplegia, ability to understand vocal cues, and ability to walk 10 m without assistance. Individuals with bilateral impairment were excluded. Conversely, inclusion criteria for the healthy group were the following: age ≥18 years, no history of injuries in the past year, and absence of musculoskeletal and neurological disorders affecting gait. The study was approved by the internal Ethical Committee and was conducted in accordance with the ethical standards of the Institute and the Declaration of World Medical Association (2013)) with its latest amendments. All participants signed an informed consent before participating in the study, with the option to withdraw from the experimental tests at any time.

### 2.2 Study design and instrumentation

The study took place from November 2021 and May 2023 in a hospital setting (Movement Analysis Laboratory of San Giuseppe Hospital), under the supervision of clinical and technical staff who managed data collection according to the experimental protocol, which involved simultaneous collection of gait trials with a marker-based optoelectronic system (MoCap) (VICON, Oxford Metrics Ltd., Oxford, United Kingdom; sampling rate: 100 Hz), and the commercial Microsoft Azure Kinect DK (MAK) RGB-Depth camera (Microsoft Inc., Redmond, WA, United States).

#### 2.2.1 Instrumented 3D gait analysis with the MoCap system

**FIGURE 1:**
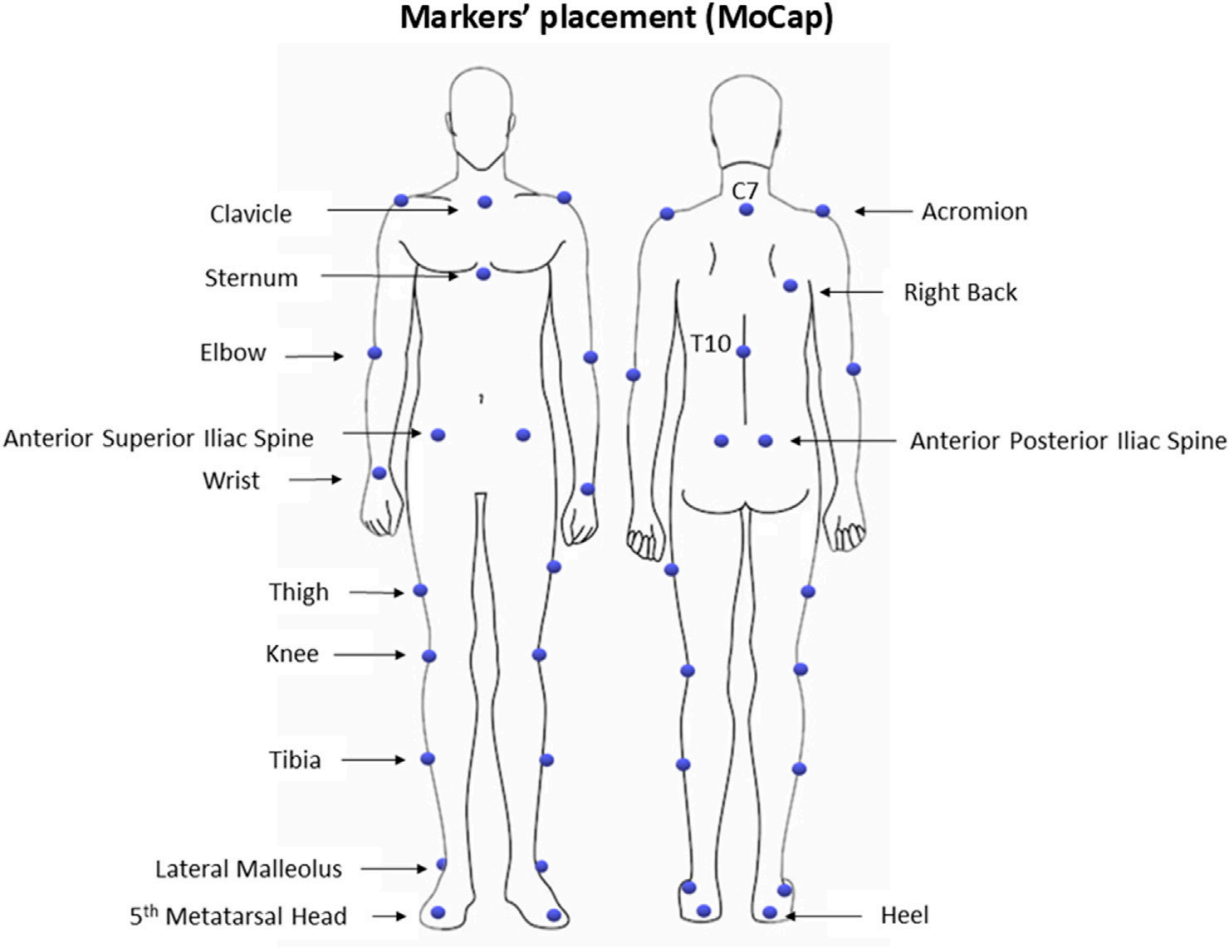
Markers’ placement for the MoCap system.

All participants underwent traditional 3D instrumental gait analysis (IGA). The laboratory space is equipped with a 6-camera MoCap system (VICON, Oxford Metrics Ltd., Oxford, United Kingdom; sampling rate: 100 Hz), two force platforms (Kistler, Winterthur, CH), and a 10 m walkway. Initially, participants’ anthropometric data were collected, including height, weight, pelvis thickness, distance between the anterior superior iliac spines, leg length, and width of knees and ankles. Then, a set of 27 spherical retro-reflective markers (Ø = 10–15 mm) was manually placed on specific anatomical landmarks of the participants’ bodies, following a modified version of the Plug-In Gait model (Davis III et al., 1991; Kadaba et al., 1990), as shown in Figure 1. The MoCap system was calibrated before each daily use according to the manufacturer’s specifications, resulting in a calibrated volume of 5 m in length (along the *x*-axis of laboratory reference system), 2 m in height (along the *y*-axis of the laboratory reference system), and 2 m in width (along the *z*-axis of the laboratory reference system).

#### 2.2.2 Motion tracking with Microsoft Azure Kinect DK

The MAK device (Microsoft Inc., Redmond, WA, United States; size: 103 × 39 × 126 mm, weight: 400 g) integrates a 1-MP time-of-flight (TOF) depth sensor (up to 1,024 × 1,024 px) and a 12-MP colour sensor (up to 4,096 × 3,072 px). The TOF technology features a global shutter with analog binning to ensure pixel synchronization during acquisition and limited noise in low-resolution operative modes (Kurillo et al., 2022). The device also incorporates a circular array of seven high-quality microphones for speech and far-field sound acquisition, and an inertial sensor (with accelerometer and gyroscope), sampled at 1.6 kHz, to manage the orientation and stability of the device. The MAK device also provides implementation of a markerless motion tracking system to capture and evaluate human body performance through the availability of depth information and a neural network-based deep learning (DL) approach: the body tracking algorithm allows 32 virtual joints of a 3D skeletal model to be estimated, which maps human body movements in real time (Liu et al., 2022).

To ensure real-time motion capture and body-tracking, the MAK device has been connected via a USB-3.0 port to a high-performance mini-pc (ZOTAC^©^, Zotac, Fo Tan, New Territories, Hong Kong, China; processor: 2.4 GHz quad-core 9^th^ generation, graphics card: NVIDIA GeForce RTX 2060 6 GB, RAM: 16 GB). Among the native operative modes, the MAK device was configured to optimize performance and ensure as accurate tracking of gait as possible. The colour stream was set to 1920 × 1080 px resolution, while the depth stream mode was set to unbanned NFOV (near field of view) to fit spatial requirements. The frame rate was set to 30 fps for both streams to fit the real-time requirement. Concerning the Software Development Kits (SDKs), version 1.4.1 was used to collect data from the device and version 1.1.0 to handle the body tracking features. In addition, an *ad hoc* wrapper was implemented to integrate both SDKs within the Unity^®^ development environment (Unity Technologies, San Francisco, CA, United States), thus creating a user-friendly solution whose graphical user interface (GUI) simplifies system management and data acquisition for operators and provides text and audio cues for patients as they walk to the device.

#### 2.2.3 Experimental setup

**FIGURE 2:**
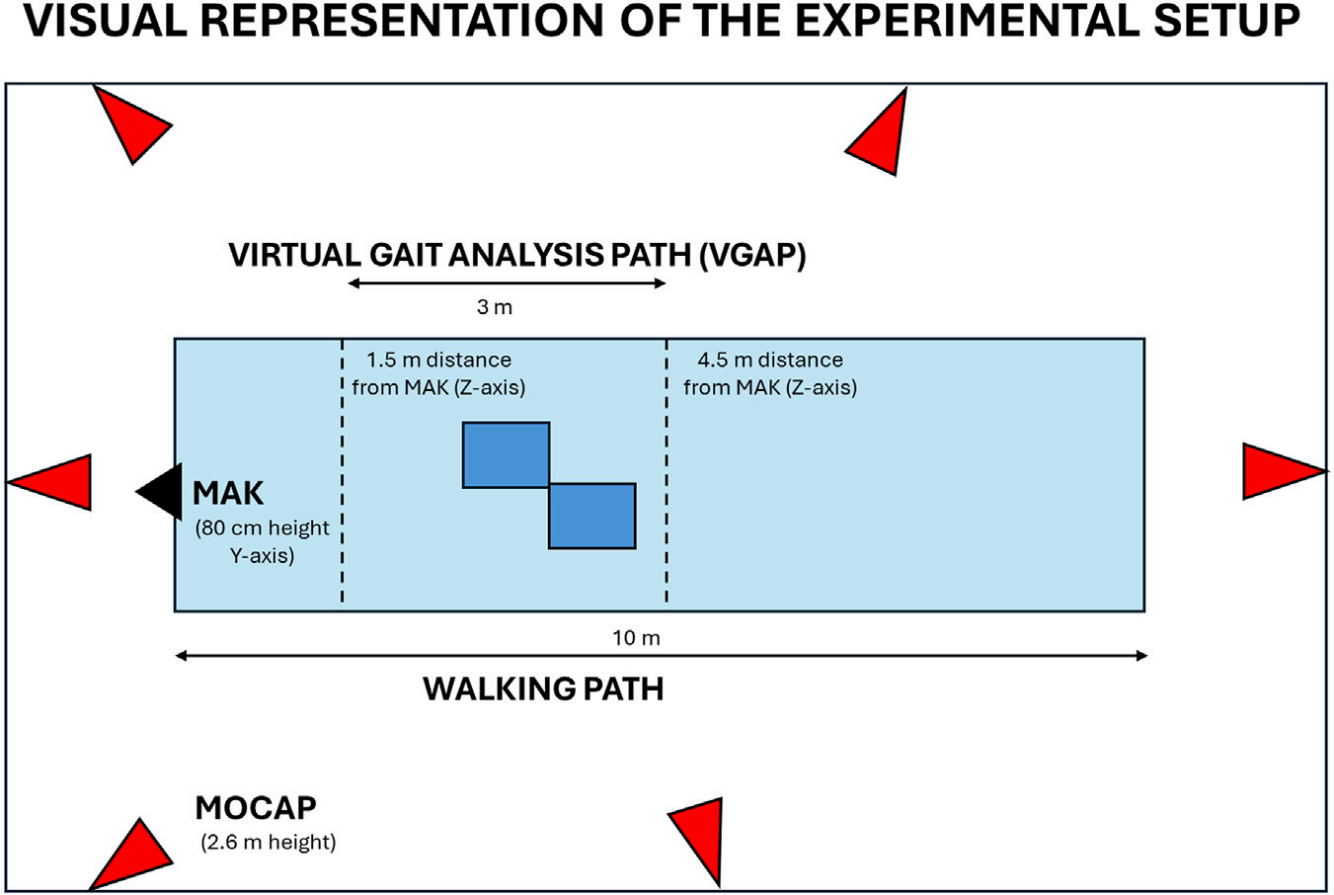
Visual representation of the experimental set up. The schema includes the position of MAK (black), the MoCap cameras (red), and the virtual gait analysis path inside the walkway (light blue) with integrated force plates (dark blue) to ensure total body motion capture and analysis.

The experimental setup was the same as that proposed in our previous validation studies (Cimolin et al., 2022; Ferraris et al., 2021) to allow a fair comparison between the gait parameters estimated by the two systems (Figure 2). The MAK device was placed on a tripod at the end of the laboratory walkway to ensure its stability and easy correction of angular orientation along the reference axes. Since a loss of accuracy can be observed in non-frontal configurations, such as rear and side views (Gianaria and Grangetto, 2019; Müller et al., 2017), the experimental setup was chosen to capture gait while walking in front of the device to maximize depth maps and the body tracking accuracy along the central cone of vision (Albert et al., 2020; Kurillo et al., 2022; Wang et al., 2015). In this setting, body tracking functions are automatically activated when the person enters the MAK’s field of view, about 5 m away, with maximum accuracy and robustness (Ferraris et al., 2021).

Since the experimental protocol involves the use of both MAK and MoCap system, a common virtual gait analysis pathway (VGAP) was defined in the central area of the laboratory walkway and around the force platforms to ensure whole-body motion capture and analysis with the two systems. The VGAP is about 3 m long, starts at 4.5 m and ends at 1.5 m from the MAK to ensure the whole-body framing even near the device. Previous work has shown that this short length is still sufficient to detect at least one full stride per leg (Eltoukhy et al., 2017) necessary to estimate gait parameters. According to the experimental protocol and IGA requirements, participants were asked to walk barefoot along the walkway at their natural pace. Up to three successful trials were collected to guarantee the reproducibility of the results in terms of gait parameters.

### 2.3 Data analysis and processing

Raw optical data collected by MoCap were processed using dedicated software for data tracking and analysis, namely, Nexus (Version 1.8, VICON, Oxford Metrics Ltd., Oxford, United Kingdom) and Polygon (Version 2.4, VICON, Oxford Metrics Ltd., Oxford, United Kingdom). Regarding MAK, the 3D trajectories of joints collected during gait and locally stored as JSON files were processed through an *ad hoc* and custom-written MATLAB^®^ (Mathworks Inc., Natick, MA, United States) script.

#### 2.3.1 Gait spatio-temporal parameters

Concerning MoCap data, the main gait events (i.e., right/left heel strike and toe off) were manually defined. In particular, gait events and cycles were identified starting from the steps performed nearby laboratory’s force plates, also corresponding to the VGAP area defined for the analysis with MAK according to the proposed experimental setup. Spatio-temporal gait parameters were then automatically computed by the previously mentioned software. Other quantities estimated during IGA (e.g., joint kinematics and kinetics) were not considered for data analysis, although available.

**FIGURE 3:**
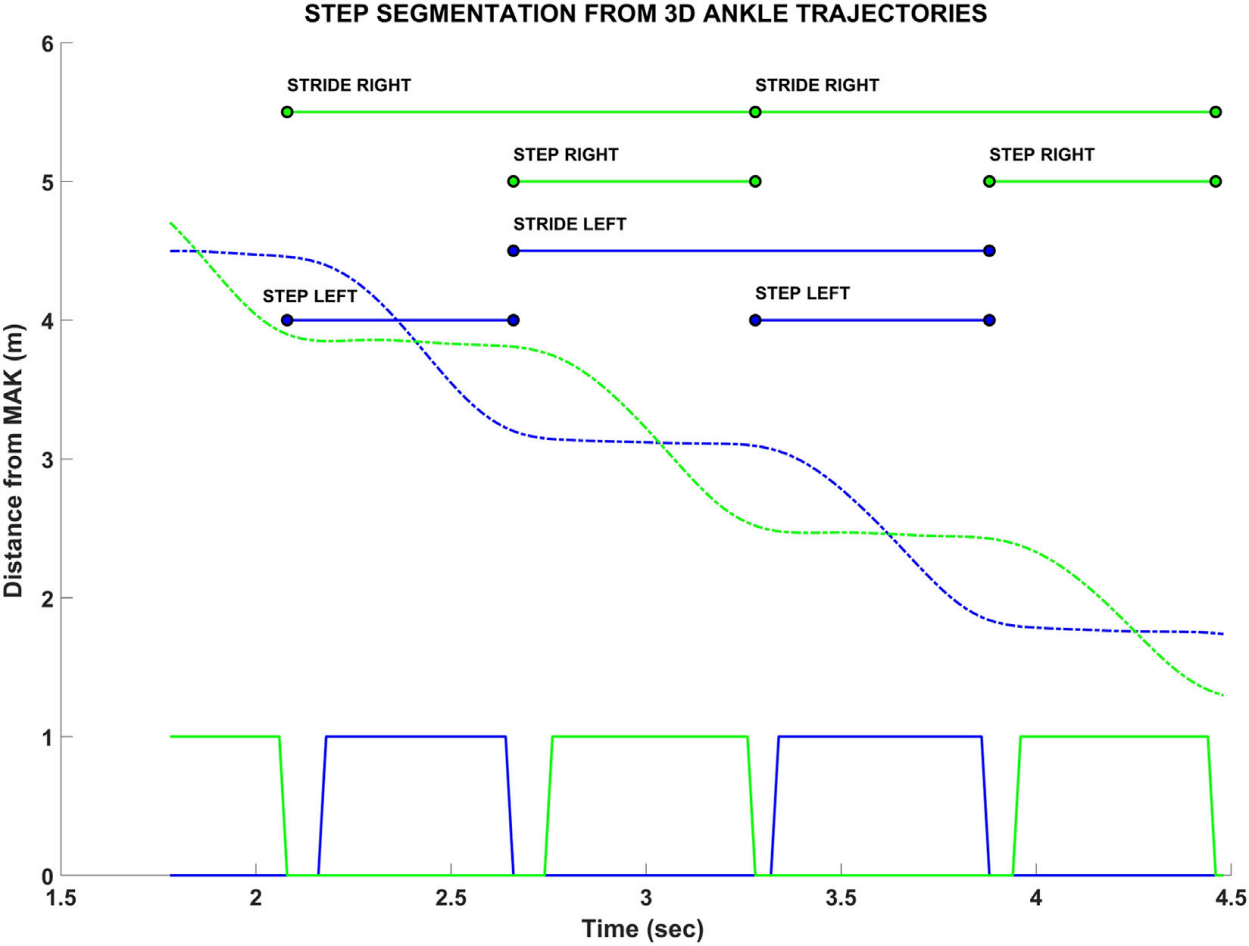
Example of the step segmentation procedure from the ankle trajectories of MAK (green and blue dashed lines for right and left ankles, respectively). The squared lines (bottom) represent the trajectories segmentation: 0 value refers to the stance (stationary) condition, 1 value refers to the swing (in motion) condition. Above, the estimated steps and strides are shown, with their initial and final instants. The corresponding spatial information, for both instants, is estimated from the joint z components (i.e., depth values).

Concerning the MAK data, the 3D joint trajectories of the skeletal model were first resampled to 50 Hz using a cubic interpolation to overcome minimal jitter in the acquisition frequency and timestamps, and to enhance the overall spatial resolution. Next, a third-order Butterworth low-pass (5 Hz) filter was applied to remove noise in the high-frequency band. After the data pre-processing phase, a step segmentation procedure was applied on the ankle trajectories: the procedure works on ankles because they show greater accuracy than feet (Gama et al., 2019; Tsai et al., 2023). Specifically, the step segmentation algorithm detects whether each ankle joint is stationary (i.e., stance phase) or in motion (swing phase) when the difference between the depth values (joint z-component) of two consecutive samples is less than or greater than a pre-defined threshold (2 cm) (Ferraris et al., 2021). From the result of the step segmentation procedure (Figure 3), it is possible to estimate all primary and derived spatio-temporal parameters related to the steps within the VGAP area.

The following primary and derived spatio-temporal gait parameters (Table 1) were considered for further analysis and comparison between the two systems. As shown in Table 1, it is important to emphasize that spatio-temporal parameters are calculated with different algorithms for the two systems because different sources of information are available. All the spatio-temporal parameters were computed separately for the right and left legs and then they were pooled together.

**TABLE 1:** List of spatio-temporal gait parameters included in the study with the meaning (i.e., computation method) for each system.

Parameter [unit]	MoCap meaning	MAK meaning
Step length [m]	Longitudinal distance from one-foot strike to the next one	Distance (difference in depth values) between the final and initial instants of one-foot step
Step time [s]	Time between two consecutive heel strikes	Time between the final and initial instants of one-foot step
Stride length [m]	Distance between two successive placements of the same foot	Distance (difference in depth values) between the final and initial instants of one-foot stride
Stride time [s]	Time elapsed between the first contact of two consecutive footsteps of the same foot	Time between the final and initial instants of one-foot stride
Double support [s]	Time in which both feet are in contact with the ground	Time inside a gait cycle where both ankles are in stationary condition
Foot off [%]	Duration of the stance phase, as % of the gait cycle	Duration of a one-foot stance phase (stationary condition), as % of gait cycle
Walking speed [m/s]	Ratio between stride length and stride time	Ratio between one-foot stride length and time
Cadence [steps/min]	Number of steps in a time unit (ratio between 120 and stride time)	Number of steps in a time unit (ratio between 120 and stride time)

#### 2.3.2 CoM parameters

**FIGURE 4:**
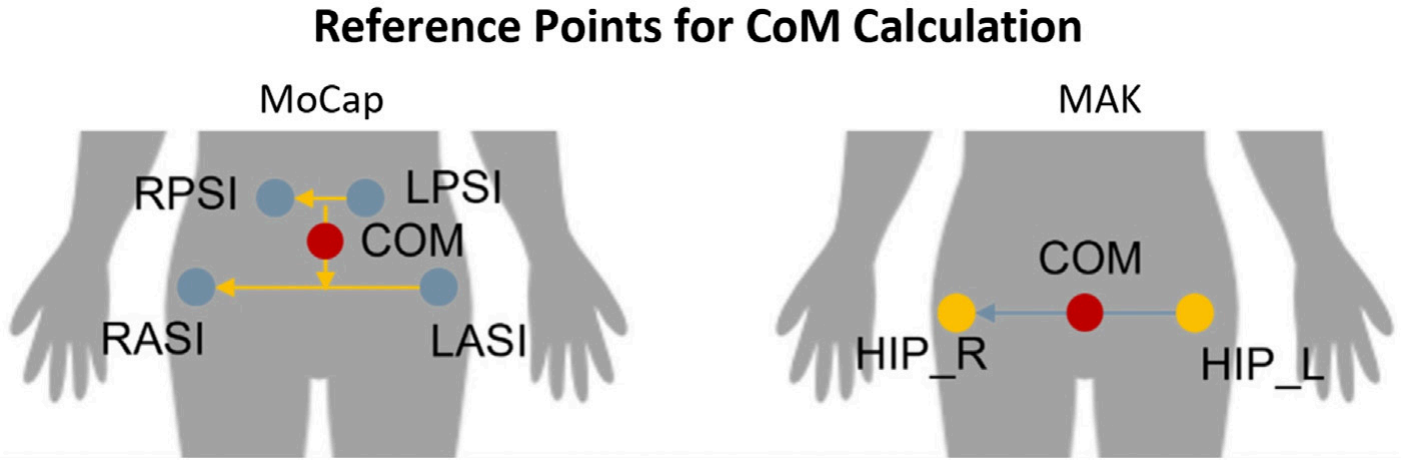
Reference points used to compute the position of the CoM with MoCap (left) and MAK (right).

Gait-related CoM parameters were computed from MoCap data using an *ad hoc* routine in SmartAnalyzer (BTS Bioengineering SPA, Milano, Italia). CoM estimation was defined with respect to laboratory reference system relying on the trajectories specific markers applied on the body. In particular, CoM was computed as the midpoint of the vector connecting the midpoints of the markers on the right and left anterior superior iliac spines, and right and left posterior superior iliac spines, respectively. Regarding MAK, the position of CoM was defined as the midpoint between the 3D hip joints, according to the MAK skeletal model. Figure 4 shows the position of CoM for the two systems.

To analyze body displacements during gait, the CoM trajectory was retrieved and its maximum excursions along the three principal directions across a complete gait cycle was computed for both systems. In particular, the following parameters were calculated:

• Medio-lateral (ML) displacement: excursion along the medio-lateral direction of gait (perpendicular to the walking direction), corresponding to the *z*-axis of the MoCap reference system and the *x*-axis of the MAK. It evaluates the maximum lateral body sway inside the gait cycles.
• Vertical (V) displacement: excursion along the vertical direction of gait (perpendicular to the walking direction), corresponding to the *y*-axis of the MoCap reference system and the *y*-axis of the MAK. It evaluates the maximum vertical body sway inside the gait cycles.
• Antero-posterior (AP) displacement: excursion of the CoM along the antero-posterior direction of gait, corresponding to the *x*-axis of the MoCap reference system and the *z*-axis of the MAK. It evaluates the progression of the body, along the walking direction, inside the gait cycles. It should be noted that such parameter does not indicate an actual sway of the CoM but its displacement along the direction of gait and it is usually not relevant for assessing the risk fall risk. However, since this is a validation study, it was included to compare the two systems in estimating such measure.

As shown in Figure 4, it is important to emphasize that CoM parameters are calculated differently for the two systems because involved markers (for MoCap) and joints (for MAK) differ in number and anatomical positions. All CoM parameters were computed for each gait cycle and then they were pooled together.

### 2.4 Statistical analysis

Statistical analysis was performed using MATLAB (version R2023a, The MathWorks Inc., Natick, MA, United States), and it was based on three consistent IGA trials of each participant. Data was checked for normality via the Kolmogorov-Smirnov test. For each parameter, paired t-test was performed to assess the presence of statistically significant differences (p < 0.05) between the measurements performed with the two systems. The mean accuracy for each parameter, expressed as a percentage, was then assessed according to the following formula (Equation 1):

$$Accuracy=100\%-\text{MAPE}=100\%-\left(mean\left(\left|\frac{{Measured\;value}_{\left(MAK\right)}-{Actual\;value}_{\left(MoCap\right)}}{{Actual\;value}_{\left(MoCap\right)}}\right|\right)\right)\;*100\;\%$$ (1)

where MAPE represents the Mean Absolute Percentage Error computed on all measured values.

Pearson’s correlation coefficient (r) was calculated to describe the agreement between the measurements retrieved from the MoCap and MAK systems. The limits of agreement (LoA) between them was also evaluated through Bland-Altman (BA) analysis, a graphical method to compare two measurements which is also useful to assess if a method overestimates high values and underestimates low values (Giavarina, 2015). In addition, the root mean square error (RMSE) was computed for each parameter according to the following equation (Equation 2):

$$RMSE=\sqrt{\sum_{i=1}^{n}\frac{{\left(\hat{y_{i}}-y_{i}\right)}^{2}}{n}}=\sqrt{\sum_{i=1}^{n}\frac{e_{i}^{2}}{n}}$$ (2)

where ŷ1 … ŷn are the values computed from the MoCap, y1 … yn are the values computed from the MAK (thus e1 … en are the errors), and n is the number of observations (i.e., number of gait trials).

Data analysis was performed by pooling together the data collected from both experimental groups (stroke and healthy subjects), and then performed separately in order to highlight any differences in performance and accuracy when measuring healthy and pathological gait, respectively.

## 3 Results

A total of 47 individuals were enrolled in the study and included in the two groups according to the inclusion criteria given in the previous section. Participants’ characteristics are resumed in Table 2.

**TABLE 2:** Mean anthropometric and clinical features of participants. Values are expressed as mean and standard deviation (SD), except for the number of participants, which is divided into males (M) and females (F) for each group.

	All	Stroke	Healthy
Participants (M/F)	47 (23/24)	20 (11/9)	27 (12/15)
Age (years)	52.86 (10.98)	63.84 (11.95)	41.87 (10.01)
Body mass (kg)	74.88 (11.17)	83.08 (11.58)	66.67 (10.76)
Height (cm)	170.31 (8.04)	169.61 (9.08)	171 (7.00)
BMI (kg/m^2^)	25.91 (3.69)	28.96 (4.12)	22.86 (3.26)

### 3.1 Gait spatio-temporal parameters

**FIGURE 5:**
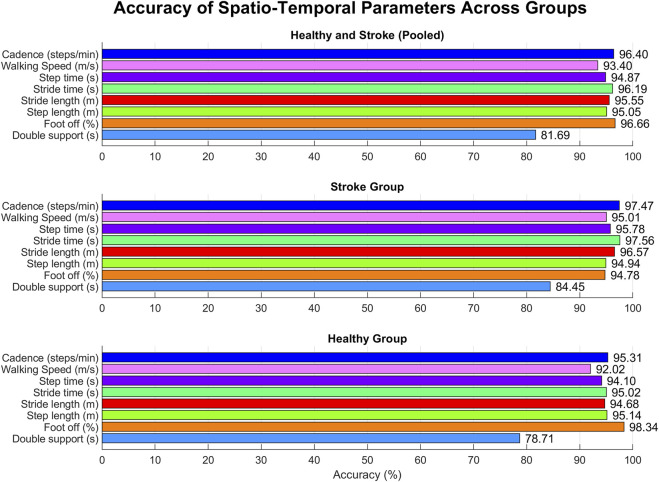
Accuracy for each spatio-temporal parameter within each group.

Kolmogorov-Smirnov test confirmed the normality of data, thus allowing for their representation in terms of mean and standard deviation. Spatio-temporal gait parameters calculated with both systems are resumed in Table 3, together with the results of the agreement analysis between MoCap and MAK. The computed parameters appear to be coherent between the two systems, as supported by the corresponding accuracy and RMSE values, and by the absence of statistically significant inter-system differences revealed by the paired t-test (p > 0.05). These considerations are further supported by the corresponding values of Pearson’s correlation coefficient (r), indicating an overall statistically significant strong correlation (p < 0.05, r > 0.7) between MoCap and MAK. Notably, the same considerations could be extended to the pooled data of healthy and stroke individuals, as well as when considering the subgroup analysis, thus suggesting no differences in performance and accuracy of MAK when dealing either with healthy or pathological gait. The only exception is the double support parameter in healthy group that shows lower and non-significant correlation (p > 0.05, r < 0.6): this may depend on the greater difficulty in deriving this very subtle temporal parameter from MAK information (both joint ankles in stationary conditions) in subjects characterized by greater joint mobility and speed. The bar charts in Figure 5 display the accuracy for each parameter within each group, enhancing visual assessment and facilitating performance comparison across groups.

**TABLE 3:** Mean and standard deviation values, p-values associated with the performed paired t-test, accuracy, Pearson’s correlation coefficients, and RMSE values for the spatio-temporal gait parameters estimated with the two systems, divided by group.

Group	Parameter	MoCap	MAK	p-value	Accuracy	r	RMSE
All	Double support (s)	0.38 (0.32)	0.41 (0.32)	0.621	81.69%	0.98*	0.07
	Foot off (%)	63.24 (5.65)	64.52 (6.52)	0.185	96.66%	0.89*	3.23
	Step length (m)	0.55 (0.18)	0.55 (0.18)	0.812	95.05%	0.98*	0.03
	Stride length (m)	1.10 (0.37)	1.08 (0.35)	0.648	95.55%	0.98*	0.07
	Stride time (s)	1.30 (0.42)	1.31 (0.38)	0.876	96.19%	0.99*	0.08
	Step time (s)	0.65 (0.21)	0.65 (0.20)	0.072	94.87%	0.98*	0.04
	Walking Speed (m/s)	0.98 (0.49)	0.93 (0.44)	0.494	93.40%	0.98*	0.11
	Cadence (steps/min)	100.12 (24.39)	98.08 (22.14)	0.589	96.40%	0.98*	5.38
Stroke	Double support (s)	0.61 (0.36)	0.64 (0.34)	0.690	84.45%	0.97*	0.09
	Foot off (%)	67.38 (5.98)	70.06 (5.75)	0.057	94.78%	0.80*	4.55
	Step length (m)	0.38 (0.11)	0.39 (0.11)	0.668	94.94%	0.98*	0.02
	Stride length (m)	0.76 (0.22)	0.75 (0.23)	0.977	96.57%	0.99*	0.03
	Stride time (s)	1.62 (0.44)	1.60 (0.40)	0.841	97.56%	0.99*	0.08
	Step time (s)	0.81 (0.21)	0.80 (0.20)	0.118	95.78%	0.98*	0.04
	Walking Speed (m/s)	0.52 (0.24)	0.52 (0.24)	0.979	95.01%	0.99*	0.03
	Cadence (steps/min)	79.07 (19.60)	79.50 (19.06)	0.926	97.47%	0.99*	2.28
Healthy	Double support (s)	0.19 (0.03)	0.21 (0.06)	0.163	78.71%	0.56	0.05
	Foot off (%)	59.71 (1.29)	59.87 (1.83)	0.651	98.34%	0.71*	1.27
	Step length (m)	0.69 (0.07)	0.70 (0.08)	0.838	95.14%	0.85*	0.04
	Stride length (m)	1.40 (0.15)	1.35 (0.15)	0.138	94.68%	0.84*	0.09
	Stride time (s)	1.02 (0.07)	1.06 (0.07)	0.817	95.02%	0.71*	0.06
	Step time (s)	0.51 (0.03)	0.53 (0.04)	0.102	94.10%	0.73*	0.04
	Walking Speed (m/s)	1.38 (0.21)	1.28 (0.19)	0.799	92.02%	0.85*	0.15
	Cadence (steps/min)	118.09 (8.39)	113.93 (7.10)	0.685	95.31%	0.74*	7.01

‘*’ = p-value <0.05 for Pearson’s correlation.

**FIGURE 6:**
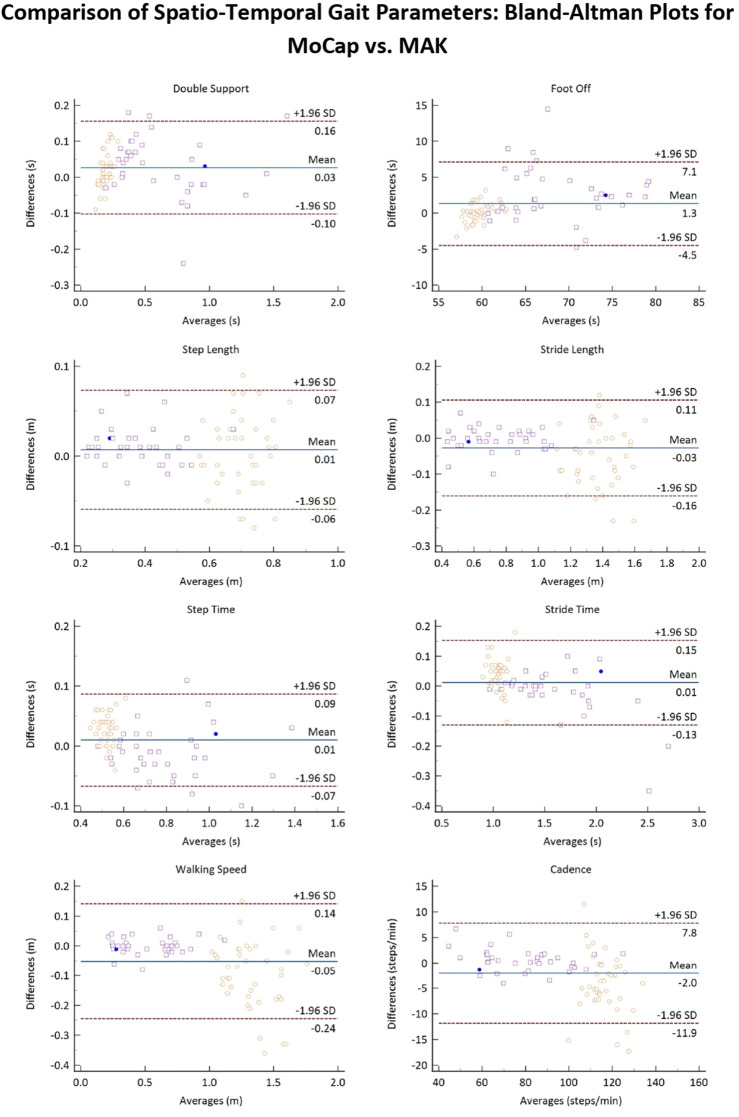
Bland-Altman plots of the average values of the spatio-temporal gait parameters retrieved from MoCap and MAK plotted against the differences for both systems. Purple squares and yellow circles denote the stroke and the healthy groups, respectively. Blue points denote correspondence between a value for both groups.

Figure 6 reports the Bland-Altman plots for the estimated spatio-temporal parameters when considering the pooled data. The horizontal lines indicate the mean difference and the limits of agreement (LoA), defined as the mean difference ±1.96*standard deviation. The differences between the two paired values are displayed as y-values, whilst their average values are reported as x-values. In the current analysis no evident bias could be observed and 95% of the differences falls inside the LoA, indicating a globally good association between MoCap and MAK, which are also coherent with the results reported in Table 3.

### 3.2 CoM parameters

**FIGURE 7:**
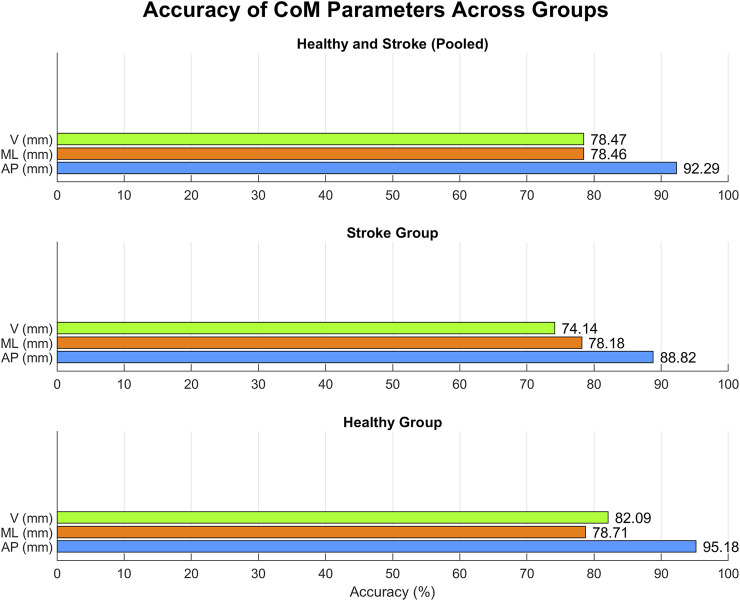
Accuracy for each CoM parameter within each group.

Gait-related CoM parameters computed for both systems are resumed in Table 4, together with the results of the statistical t-test (shown in the MAK column) and of the agreement analysis. Except for ML excursion in healthy individuals, an overall statistically strong correlation (p < 0.05, r > 0.7) can be observed for all parameters, either considering the pooled data or the subgroups. Figure 7 presents the accuracy of gait-related CoM parameters across different groups, similarly to Figure 5.

**TABLE 4:** Mean and standard deviation values, p-values associated with the performed paired t-test, accuracy, Pearson’s correlation coefficients, and RMSE values for gait-related CoM parameters estimated with the two systems, divided by group.

Group	Parameter	MoCap	MAK	p-value	Accuracy	r	RMSE
All	AP (mm)	1,095.05 (365.96)	1,103.28 (337.26)	0.886	92.29%	0.97*	90.71
	ML (mm)	64.70 (31.20)	57.92 (21.12)	0.119	78.46%	0.88*	17.32
	V (mm)	37.41 (14.09)	36.35 (12.06)	0.619	78.47%	0.79*	8.68
Stroke	AP (mm)	746.84 (221.38)	797.62 (232.47)	0.360	88.82%	0.93*	98.14
	ML (mm)	91.41 (27.19)	72.89 (22.63)	0.003	78.18%	0.86*	23.18
	V (mm)	28.35 (13.03)	33.22 (13.58)	0.136	74.14%	0.89*	7.86
Healthy	AP (mm)	1,385.22 (139.47)	1,358.01 (144.06)	0.387	95.18%	0.84*	84.2
	ML (mm)	42.43 (8.83)	45.45 (6.98)	0.090	78.71%	0.28	10.09
	V (mm)	44.96 (9.85)	38.96 (9.90)	0.007	82.09%	0.74*	9.32

‘*’ = p-value <0.05 for Pearson’s correlation.

**FIGURE 8:**
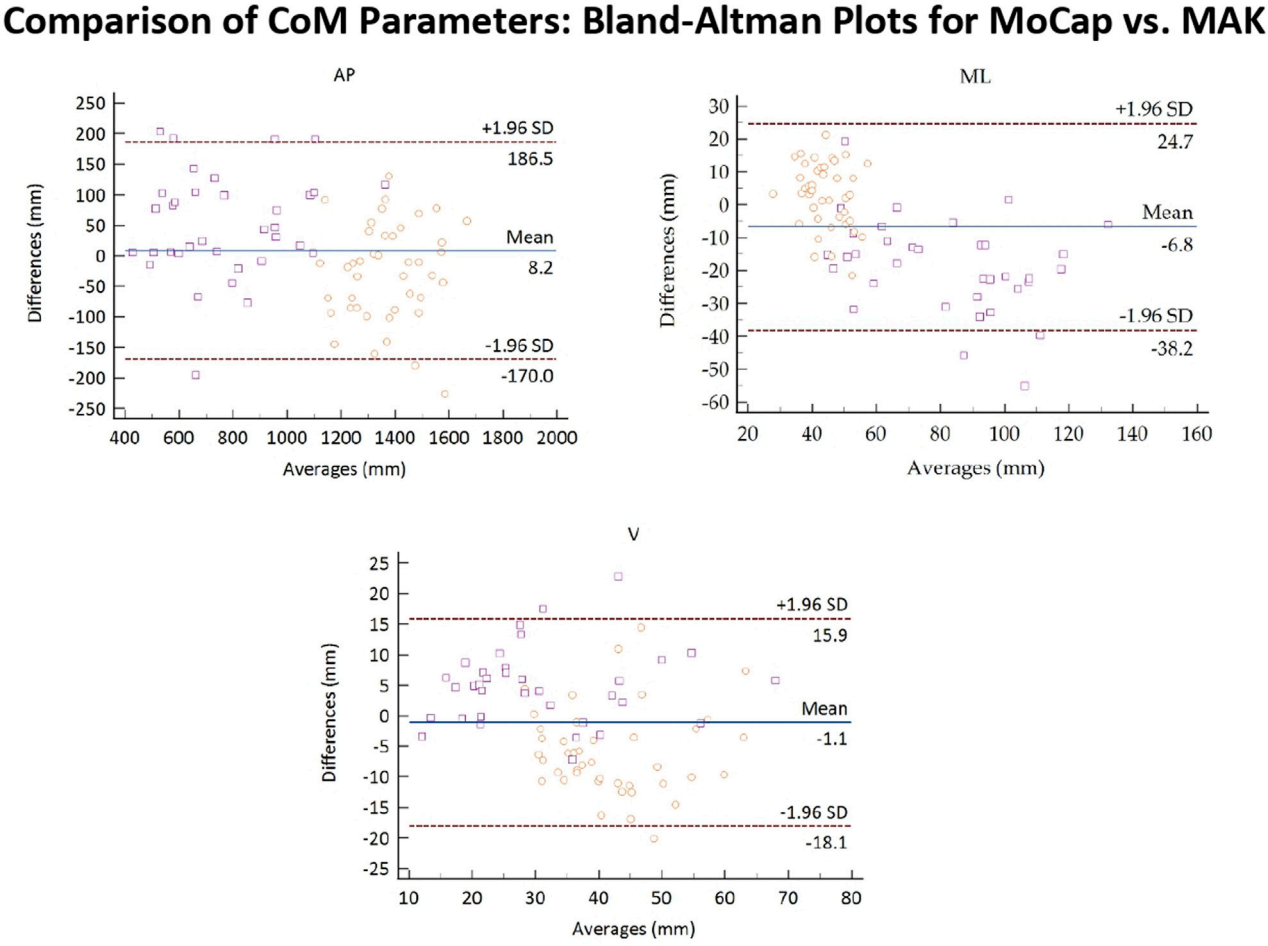
Bland-Altman plots of the average values of the CoM parameters retrieved from MoCap and MAK plotted against the differences for both systems. Purple squares and yellow circles denote the stroke and the healthy groups, respectively.

Figure 8 reports the Bland-Altman plots for the estimated CoM parameters when considering the pooled data. Also in this case, a globally good association between the two systems can be observed since 95% of the differences falls inside the LoA, and there is no evident bias. However, CoM data presents a higher dispersion with respect to gait spatio-temporal parameters, which is particularly evident not only for ML displacement in healthy subjects, but also for V displacement in both healthy and stroke groups. In particular, the V displacement appears underestimated for healthy subjects, and slightly overestimated for stroke individuals. This confirms the results shown in Table 4. In general, the slightly lower performance in accuracy and a couple of significant differences between the two systems could depend on the different estimation methods for the CoM positions. The CoM position estimated for MoCap is definitely more accurate, as it is computed from markers placed on the ventral and dorsal sides of the body. The CoM position for MAK is estimated from a pair of frontal points. The two CoMs may be therefore solicited differently during walking, resulting in different overall excursions in the three directions.

## 4 Discussion

Optoelectronic MoCap systems are acknowledged as the gold standard to quantitatively evaluate human gait, offering a comprehensive and accurate description of an individual’s movement, and enabling the identification of pathological patterns. Despite their high accuracy and reliability, MoCap systems are not suitable for large-scale screening due to their high cost, complexity, and reliance on dedicated laboratory settings (Horak et al., 2015). Over the past two decades, various alternative technologies, such as wearable devices and 2D/3D optical sensors, have emerged as complementary tools to MoCap systems. Among these, low-cost RGB-Depth cameras with body tracking algorithms have shown particular promise for the non-invasive assessment of gait, posture, and balance in both healthy and pathological conditions. However, prior to their effective integration into everyday clinical practice for instrumental motor assessment, these technologies need to be validated against gold standard systems. In this study, the concurrent validity and accuracy of the latest model of the Microsoft Kinect, the Azure Kinect (MAK), were assessed against a MoCap system in estimating gait-related spatio-temporal and CoM parameters in healthy individuals and in a group of post-stroke patients with hemiplegia.

The overall results indicate a high correlation and agreement between the parameters estimated by both systems across the study groups. Despite similar accuracy levels, spatio-temporal parameters derived from hemiplegic patients showed a stronger correlation between MoCap and MAK compared to those obtained from healthy individuals. Specifically, within the stroke group, most parameters showed a robust positive and significant correlation (r > 0.9, p < 0.05), with only the correlation of the foot-off parameter falling below 0.9. Similarly, within the healthy group, most parameters showed strong positive correlations (r > 0.7, p < 0.05), albeit with slightly lower values on average compared to those in the stroke group. The only exception was the double support parameter, which showed a moderate rather than strong correlation. These findings are consistent with previous studies, which also reported higher correlation between MoCap and MAK compared to earlier device models (Kurillo et al., 2022; Tölgyessy et al., 2021). However, direct comparisons with prior studies are limited due to variations in subject demographics, experimental setups, and the algorithms used for step segmentation and parameters estimation. Moreover, no previous studies have used MAK to estimate spatio-temporal gait parameters in pathological conditions such as hemiplegia. Regarding hemiplegic gait patterns, the agreement between spatio-temporal parameters estimated by both systems was higher than those reported in the literature on subjects with similar demographics but using previous Kinect models (Cimolin et al., 2022; Ferraris et al., 2021).

In contrast, more studies are available in the literature focusing on healthy individuals. In (Guess et al., 2022), the authors evaluated the agreement between MAK and MoCap for a limited subset of parameters, including stride length, stride time, and step length. They reported stronger correlations (r = 0.89–0.98) than those observed in our healthy group but lower than those in our stroke group. Conversely, in (Xu et al., 2015) using a previous version of the Kinect device, the authors reported generally lower correlations with MoCap for short temporal phases of gait cycles (e.g., foot-off, double support, and swing time), particularly at higher speeds. However, it is important to note that they employed a treadmill-based experimental setup to control gait speeds, which may have influenced the estimation of other gait parameters. Our findings for the healthy group are consistent with this study, as the lowest correlations were observed for sub-phases of the gait cycle (e.g., foot-off and double support), although with higher values than those reported in (Xu et al., 2015). In contrast, we observed slightly lower correlations for step time and stride time parameters, which may be attributed to the higher average walking speed of our healthy group (1.38 m/s vs. 1.07 m/s). Similarly, in a study on polyneuropathy patients (Vilas-Boas et al., 2019), the lowest correlations (r < 0.9) were associated with parameters related to sub-phases of the gait cycle (e.g., double support duration, single stance duration, and swing duration), further supporting the previously described trend. Compared to (Vilas-Boas et al., 2019) our results for the pathological group (stroke) showed lower correlations (r < 0.9) only for the foot-off parameter, while the others, including double support duration, exhibited higher correlations (r > 0.95). The reasons for this outcome are likely related to the inherent limitations of accurately estimating ground detachment events using the markerless skeletal model provided by an optical sensor compared to the more complex biomechanical models used in MoCap systems. This limitation may become more pronounced when estimating short-duration temporal parameters, particularly those associated with the gait cycle sub-phases, and is further exacerbated at higher walking speeds. This may also explain the better results obtained for the pathological groups, whose average walking speed is significantly lower than in healthy individuals. However, addressing the potential issue related to higher walking speeds in dedicated future studies could help improve the understanding of the MAK device’s performance and establish optimal requirements, settings, and applications for different operating conditions.

Regarding the results on CoM displacements during gait, both healthy and stroke groups showed strong agreement along the vertical and antero-posterior directions (r > 0.74). However, the two systems exhibited poor correlation in estimating CoM displacement along the medio-lateral direction in the healthy group (r = 0.28). This discrepancy may be due to the greater stability of healthy individuals during walking, which results in minimal lateral body oscillations compared to post-stroke individuals. Hemiplegic gait patterns are typically characterized by lateral body shifts to compensate for motor deficits due to unilateral weakness and abnormal torso tilting (Aprile et al., 2006; Takashima et al., 2016). In contrast, the small excursions and low variability observed in the healthy group likely explain the poor correlation, despite a measurement accuracy exceeding 78% and a low residual error (approximately 1 cm). Conversely, in the stroke group, the agreement between MAK and MoCap in estimating medio-lateral (r = 0.86, p < 0.05) and vertical (r = 0.89, p < 0.05) CoM excursions is higher than that reported in other studies using earlier Kinect models (Cimolin et al., 2022; Ferraris et al., 2021). This supports the device’s ability to detect effective motor pattern alterations associated with lateral displacements. However, CoM parameters showed slightly lower correlations and measurement accuracy compared to the spatio-temporal parameters. It is also important to note, as shown in Figure 3, that the positions of CoM for both systems are not identical, resulting in different solicitations during gait that may have affected the results. Nonetheless, the overall correlation and measurement accuracy values remain high (>0.79 and >78%, respectively).

In general, the findings of the present study suggest that the MAK device has the potential to provide an accurate and reliable estimation of gait-related parameters measured over path lengths that are more suitable for non-laboratory settings, where MoCap systems are not applicable, as they typically operate on larger, dedicated spaces. However, the controlled laboratory environment used in this study may differ from real-world conditions, where factors such as obstacles, variable surfaces, and lighting can affect overall performance. While the results are promising in controlled settings, further investigation is needed in unsupervised environments, such as private homes, to fully assess the applicability of MAK sensors for remote monitoring of gait patterns and rehabilitation purposes.

Furthermore, there are some limitations linked to both study populations and technological aspects that should be considered. In particular, the small number of participants for both groups limits to some extent the strength and the generalizability of the statistical results and general findings. However, it should be noted that large experimental samples are difficult to be recruited and, also, that traditional instrumented gait analysis requires subjects to wear minimal clothing, usually only underwear: this can cause embarrassment and be an issue for subjects with dysfunctions in dressing (Cerfoglio et al., 2022). In terms of technological aspects, the main critical issue could be linked to the need for better adaptation of the algorithms used for step identification and parameter estimation by the two systems. Although they provide similar information, the anatomical landmarks used to track body movements differ because the biomechanical models are different. Specifically, the MoCap system relies on the 3D coordinates of skin-mounted markers placed on specific *repere* points, while the MAK body tracking algorithm depends on virtual joint estimation of the skeletal model without any physical markers. Consequently, the different positions of these reference points may introduce some biases when estimating specific metrics, as each model relies on different body geometry and joint dynamics. These differences could be reflected in the final measurements of the parameters derived from a standard complex model (i.e., MoCap) and a simpler one (i.e., MAK).

Regarding the higher levels of agreement observed in the stroke group compared to the healthy group, this finding does not seem to have been reported in previous studies. The different results may be attributed to the sensitivity of the step segmentation algorithm; however, some bias may also arise from the limited sample size, which may affect data variability and the overall strength of the findings. One objective factor that might explain this result is the walking speed, which is typically lower in pathological subjects than in healthy individuals and that may lead to slightly less accurate parameter measurements when it increases: this seems to be mainly reflected in temporal parameters related to sub-phases of the gait cycle, as also pointed out by several previous studies. However, further investigation on wider reference samples is needed to support and verify this hypothesis.

In addition, it should be noted that the present study focused exclusively on spatio-temporal aspects of gait, while gait-related joint angles were not assessed. According to the literature, despite generally good agreement, accuracy, and correlation between the spatio-temporal parameters estimated by the two systems, Kinect-based estimation may exhibit limited accuracy and sensitivity regarding kinematic parameters such as joint angles during walking (Cerfoglio et al., 2022; Latorre et al., 2019). Given that the available literature on Kinect-based kinematics is primarily centered on the previous versions of the device, it would be interesting to evaluate how the new generation of the device performs in estimating these more complex metrics in both healthy and pathological populations.

Despite the discussed limitations of the study, the proposed gait analysis setup, based on a single Microsoft Azure Kinect DK device, demonstrates good concurrent validity compared to the gold standard system, in terms of correlation, accuracy, and residual error on both healthy and pathological groups. Its potential adoption in everyday practice could introduce new possibilities for motion analysis in various contexts. Although the strengths and weaknesses of these optical approaches require further investigation, these devices offer a non-invasive, cost-effective, and user-friendly solution for motion analysis in environments where MoCap systems are not feasible, such as outpatient settings and private home environments. This opens new perspectives for remote monitoring and rehabilitation strategies, especially in unsupervised settings. These aspects are indeed crucial for more frequent monitoring of motor performance, enabling prompt adjustments and adaptations of home-based rehabilitation programs, reducing the need for access to hospital facilities, and ultimately improving patients’ quality of life.

## 5 Conclusion

The aim of this study was to assess the concurrent validity of spatio-temporal and CoM gait-related parameters estimated through Microsoft Azure Kinect DK compared to a gold standard system for motion capture and analysis. The investigation focused on both normal and pathological gait patterns, involving hemiplegic (post-stroke survivors) and healthy subjects. Despite a few limitations linked to sample size, differences in biomechanical models and parameter computation algorithms, this device is able to provide an accurate estimation of the parameters considered, which closely match those retrieved from the gold standard for both healthy and hemiplegic individuals. Although further advancements are necessary before its full employment in clinical settings, the reported results demonstrate the usefulness and reliability of the proposed approach for quantitative gait assessment, especially in pathological populations. In addition to increasing the sample size and improving the alignment of models and algorithms, future analyses could incorporate Kinect-based assessment of gait kinematics against the gold standard, offering a more comprehensive description of an individual’s normal and pathological gait and opening new perspectives for both clinical practice and remote assessment.

## Data Availability

The datasets presented in this study can be found in online repositories. The names of the repository/repositories and accession number(s) can be found below: https://doi.org/10.5281/zenodo.11580766.
